# 3-Ethyl-1*H*-1,2,4-triazole-5(4*H*)-thione

**DOI:** 10.1107/S1600536812021927

**Published:** 2012-05-19

**Authors:** Bi Jing, Yuao-Chao Du, Ai-Xin Zhu

**Affiliations:** a Faculty of Chemistry and Chemical Engineering, Yunnan Normal University, Kunming 650504, People’s Republic of China

## Abstract

The mol­ecule of the title compound, C_4_H_7_N_3_S, exists as the thione tautomer in the solid state. The asymmetric unit consits of one mol­ecule in which all atoms are located on a crystallographic mirror plane. In the crystal, adjacent mol­ecules are linked by N—H⋯N and N—H⋯S hydrogen bonds into chains running along the *a* axis. π–π stacking inter­actions between the triazole rings [centroid–centroid distance = 3.740 (1) Å and inter­planar distance = 3.376 Å] may further stabilize the structure.

## Related literature

For applications of thione-substituted triazoles and its derivatives in coordination chemistry, see: Shivarama *et al.* (2006 ▶); Wujec *et al.* (2007 ▶); Ghassemzadeh *et al.* (2008 ▶); Zhang *et al.* (2008 ▶). For crystal structure reports of 3-(alkyl or ar­yl)-1,2,4-triazole-5-thione compounds, see: Buzykin *et al.* (2008 ▶); Pachuta-Stec *et al.* (2009 ▶). For related structures of thione-substituted 1,2,4-triazole compounds, see: Kajdan *et al.* (2000 ▶). For the previous synthesis of the title compound, see: Jones & Ainsworth (1955 ▶).
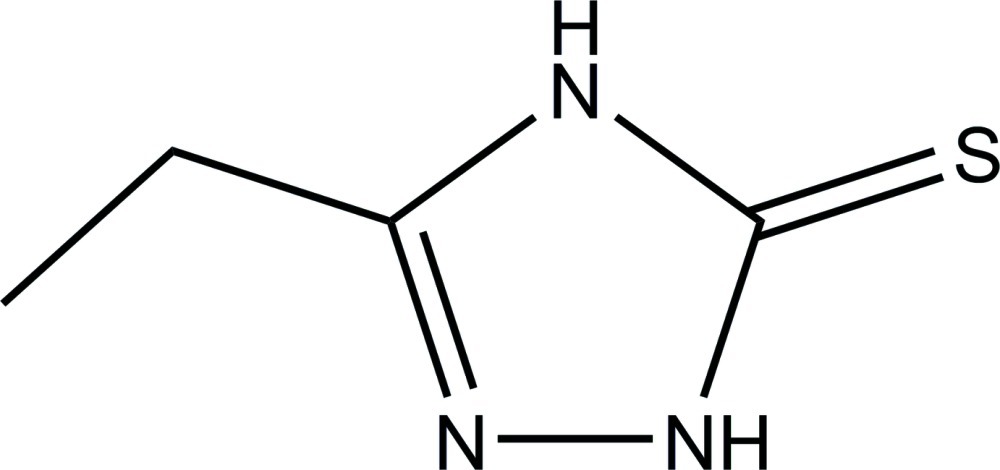


## Experimental

### 

#### Crystal data

C_4_H_7_N_3_S
*M* *_r_* = 129.19
Monoclinic, 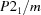

*a* = 5.0922 (10) Å
*b* = 6.7526 (14) Å
*c* = 8.6578 (17) Å
β = 90.17 (3)°
*V* = 297.70 (10) Å^3^
*Z* = 2
Mo *K*α radiation
μ = 0.43 mm^−1^
*T* = 293 K
0.26 × 0.21 × 0.11 mm

#### Data collection

Rigaku R-AXIS RAPID IP diffractometer
Absorption correction: multi-scan (*ABSCOR*; Higashi, 1995 ▶) *T*_min_ = 0.896, *T*_max_ = 0.954
2467 measured reflections
637 independent reflections
590 reflections with *I* > 2σ(*I*)
*R*_int_ = 0.034

#### Refinement

*R*[*F* ^2^ > 2σ(*F* ^2^)] = 0.042
*wR*(*F* ^2^) = 0.116
*S* = 1.09
637 reflections
49 parameters
H-atom parameters constrained
Δρ_max_ = 0.50 e Å^−3^
Δρ_min_ = −0.30 e Å^−3^

### 

Data collection: *RAPID-AUTO* (Rigaku, 1998 ▶); cell refinement: *RAPID-AUTO*; data reduction: *CrystalClear* (Rigaku/MSC, 2002 ▶); program(s) used to solve structure: *SHELXS97* (Sheldrick, 2008 ▶); program(s) used to refine structure: *SHELXL97* (Sheldrick, 2008 ▶); molecular graphics: *DIAMOND* (Brandenburg, 1999 ▶); software used to prepare material for publication: *SHELXTL* (Sheldrick, 2008 ▶).

## Supplementary Material

Crystal structure: contains datablock(s) I, global. DOI: 10.1107/S1600536812021927/nc2279sup1.cif

Structure factors: contains datablock(s) I. DOI: 10.1107/S1600536812021927/nc2279Isup2.hkl

Supplementary material file. DOI: 10.1107/S1600536812021927/nc2279Isup3.cdx

Supplementary material file. DOI: 10.1107/S1600536812021927/nc2279Isup4.cml

Additional supplementary materials:  crystallographic information; 3D view; checkCIF report

## Figures and Tables

**Table 1:** Hydrogen-bond geometry (Å, °)

*D*—H⋯*A*	*D*—H	H⋯*A*	*D*⋯*A*	*D*—H⋯*A*
N1—H1*D*⋯S1^i^	0.86	2.50	3.270 (2)	150
N3—H3*A*⋯N2^ii^	0.86	2.08	2.914 (3)	162

Symmetry codes: (i) 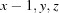; (ii) 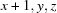.

**Fig. 1.**
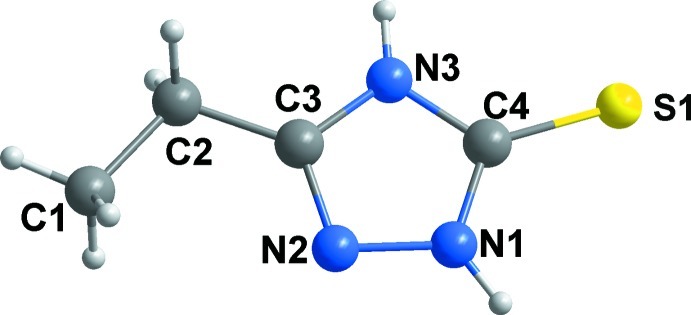
The molecular structure of the title compound, with atom labels and 30% probability displacement ellipsoids for non-H atoms.

**Fig. 2.**
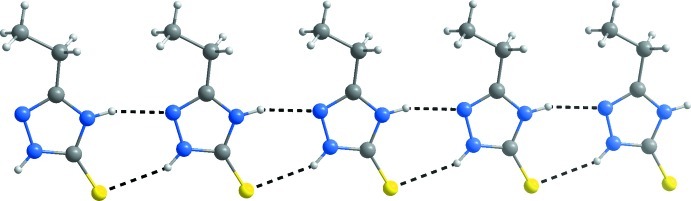
View of a chain showing the hydrogen bonding interactions as dashed lines.

## supplementary crystallographic information

### Comment

Thione-substituted triazoles have attracted increasing attention as important class of N, S-donor ligands owing to biological activity as well as the coordination properties combing heterocyclic nitrogen and exocyclic sulfur donor atoms for the construction of novel mononuclear, polynuclear, and multi-dimensional triazolate coordination compounds with interesting optical properties (Shivarama *et al.* 2006; Wujec *et al.* 2007; Ghassemzadeh *et al.* 2008; Zhang *et al.* 2008). Although there are many crystal structure of thione-substituted 1,2,4-triazoles compounds reported in the literature, most of them are based on 4-amino 3-(aryl or alkyl)-1,2,4-triazole-5-thione. Up to now, there are only a few crystal structure reports of 3-(alkyl or aryl)-1,2,4-triazole-5-thione compounds (Buzykin *et al.*2008; Pachuta-Stec *et al.* 2009). Herein we report the synthesis and the crystal structure of the title compound.

The title molecule exists as the thione tautomer in the solid state (Fig. 1), with the H atom H3 at the nitrogen adjacent to the C—S group. The bond lengths and angles are comparable to that reported in related compounds (Kajdan *et al.* 2000). All atoms of the title compound are lcoated on a crystallographic mirror plane and therefore, the molecule is planar. In the crystal structure the molecules are linked by Adjacent molecules are linked by intermolecular N—H···N and N—H···S hydrogen bonding into chains that are running along the crystallographic *a* axis (Fig. 2 and Table 1)). There are pi-pi stacking interactions between the triazole rings of neighbouring chains (centroid-centroid distance = 3.740 (1) Å, interplanar distance 3.376 Å) which may further stabilize the structure.

### Experimental

The ligand 3-ethyl-1*H*-1,2,4-triazole-5(4*H*)-thione was synthesized according to the literature method (Jones & Ainsworth 1955). A mixture of 3-ethyl-1*H*-1,2,4-triazole-5(4*H*)-thione (12.9 mg, 0.1 mmol) and water (5 ml) was placed in a Teflon-lined stainless steel vessel (15 ml) and heated at 413 K for 48 h and then cooled to room temperature at a rate of 5 K h-1. From the resulting colorless solution the solvent was slowly evaporated in air for over a week which results in the formation of colorless rod like crystals of the title compound suitable for single crystal X-ray diffraction.

### Refinement

All H atoms were located in difference map but were placed in idealized positions (N—H = 0.86 Å and C—H = 0.96-0.97 Å) and refined as riding atoms with *U*_iso_(H) = 1.2*U*_eq_(C, N) (1.5 for methyl H atoms).

### Crystal data

**Table:**

C_4_H_7_N_3_S	*Z* = 2
*M**_r_* = 129.19	*F*(000) = 136
Monoclinic, *P*2_1_/*m*	*D*_x_ = 1.441 Mg m^−^^3^
Hall symbol: -P 2yb	Mo *K*α radiation, λ = 0.71073 Å
*a* = 5.0922 (10) Å	µ = 0.43 mm^−^^1^
*b* = 6.7526 (14) Å	*T* = 293 K
*c* = 8.6578 (17) Å	Rod, colorless
β = 90.17 (3)°	0.26 × 0.21 × 0.11 mm
*V* = 297.70 (10) Å^3^	

### Data collection

**Table:**

Rigaku R-AXIS RAPID IP diffractometer	637 independent reflections
Radiation source: fine-focus sealed tube	590 reflections with *I* > 2σ(*I*)
Graphite monochromator	*R*_int_ = 0.034
ω scans	θ_max_ = 26.0°, θ_min_ = 3.8°
Absorption correction: multi-scan (*ABSCOR*; Higashi, 1995)	*h* = −6→6
*T*_min_ = 0.896, *T*_max_ = 0.954	*k* = −8→7
2467 measured reflections	*l* = −10→9

### Refinement

**Table:**

Refinement on *F*^2^	Primary atom site location: structure-invariant direct methods
Least-squares matrix: full	Secondary atom site location: difference Fourier map
*R*[*F*^2^ > 2σ(*F*^2^)] = 0.042	Hydrogen site location: inferred from neighbouring sites
*wR*(*F*^2^) = 0.116	H-atom parameters constrained
*S* = 1.09	*w* = 1/[σ^2^(*F*_o_^2^) + (0.0332*P*)^2^ + 0.4796*P*] where *P* = (*F*_o_^2^ + 2*F*_c_^2^)/3
637 reflections	(Δ/σ)_max_ < 0.001
49 parameters	Δρ_max_ = 0.50 e Å^−^^3^
0 restraints	Δρ_min_ = −0.30 e Å^−^^3^

### Special details

**Table:**

Geometry. All e.s.d.'s (except the e.s.d. in the dihedral angle between two l.s. planes) are estimated using the full covariance matrix. The cell e.s.d.'s are taken into account individually in the estimation of e.s.d.'s in distances, angles and torsion angles; correlations between e.s.d.'s in cell parameters are only used when they are defined by crystal symmetry. An approximate (isotropic) treatment of cell e.s.d.'s is used for estimating e.s.d.'s involving l.s. planes.
Refinement. Refinement of *F*^2^ against ALL reflections. The weighted *R*-factor *wR* and goodness of fit *S* are based on *F*^2^, conventional *R*-factors *R* are based on *F*, with *F* set to zero for negative *F*^2^. The threshold expression of *F*^2^ > σ(*F*^2^) is used only for calculating *R*-factors(gt) *etc*. and is not relevant to the choice of reflections for refinement. *R*-factors based on *F*^2^ are statistically about twice as large as those based on *F*, and *R*- factors based on ALL data will be even larger.

### Fractional atomic coordinates and isotropic or equivalent isotropic displacement parameters (Å^2^)

**Table:**

	*x*	*y*	*z*	*U*_iso_*/*U*_eq_	Occ. (<1)
S1	0.45391 (12)	0.2500	0.74979 (7)	0.03824 (18)	
N1	0.0168 (4)	0.2500	0.5691 (3)	0.0384 (6)	
H1D	−0.0890	0.2500	0.6464	0.046*	
N2	−0.0665 (4)	0.2500	0.4165 (2)	0.0344 (5)	
N3	0.3624 (4)	0.2500	0.4359 (2)	0.0347 (5)	
H3A	0.5244	0.2500	0.4082	0.042*	
C1	−0.0885 (5)	0.2500	0.0845 (3)	0.0447 (8)	
H1A	−0.0616	0.2500	−0.0252	0.067*	
H1B	−0.1855	0.3661	0.1135	0.067*	0.50
H1C	−0.1855	0.1339	0.1135	0.067*	0.50
C2	0.1729 (5)	0.2500	0.1657 (3)	0.0377 (7)	
H2A	0.2710	0.1340	0.1336	0.045*	0.50
H2B	0.2710	0.3660	0.1336	0.045*	0.50
C3	0.1532 (5)	0.2500	0.3376 (3)	0.0311 (6)	
C4	0.2769 (5)	0.2500	0.5841 (3)	0.0345 (6)	

### Atomic displacement parameters (Å^2^)

**Table:**

	*U*^11^	*U*^22^	*U*^33^	*U*^12^	*U*^13^	*U*^23^
S1	0.0270 (3)	0.0578 (4)	0.0299 (3)	0.000	−0.0018 (2)	0.000
N1	0.0287 (10)	0.0526 (14)	0.0338 (11)	0.000	0.0008 (9)	0.000
N2	0.0258 (9)	0.0435 (12)	0.0339 (11)	0.000	−0.0006 (8)	0.000
N3	0.0204 (9)	0.0486 (13)	0.0349 (11)	0.000	0.0017 (8)	0.000
C1	0.0332 (13)	0.0639 (19)	0.0368 (14)	0.000	−0.0086 (11)	0.000
C2	0.0276 (11)	0.0535 (16)	0.0321 (12)	0.000	−0.0014 (10)	0.000
C3	0.0229 (10)	0.0334 (13)	0.0368 (12)	0.000	−0.0025 (9)	0.000
C4	0.0274 (11)	0.0368 (13)	0.0393 (13)	0.000	0.0003 (10)	0.000

### Geometric parameters (Å, º)

**Table:**

S1—C4	1.692 (3)	C1—C2	1.504 (4)
N1—C4	1.330 (3)	C1—H1A	0.9600
N1—N2	1.386 (3)	C1—H1B	0.9600
N1—H1D	0.8600	C1—H1C	0.9600
N2—C3	1.312 (3)	C2—C3	1.492 (4)
N3—C4	1.356 (3)	C2—H2A	0.9700
N3—C3	1.362 (3)	C2—H2B	0.9700
N3—H3A	0.8600		
			
C4—N1—N2	113.2 (2)	C3—C2—C1	113.8 (2)
C4—N1—H1D	123.4	C3—C2—H2A	108.8
N2—N1—H1D	123.4	C1—C2—H2A	108.8
C3—N2—N1	103.7 (2)	C3—C2—H2B	108.8
C4—N3—C3	109.8 (2)	C1—C2—H2B	108.8
C4—N3—H3A	125.1	H2A—C2—H2B	107.7
C3—N3—H3A	125.1	N2—C3—N3	109.9 (2)
C2—C1—H1A	109.5	N2—C3—C2	125.4 (2)
C2—C1—H1B	109.5	N3—C3—C2	124.6 (2)
H1A—C1—H1B	109.5	N1—C4—N3	103.3 (2)
C2—C1—H1C	109.5	N1—C4—S1	127.6 (2)
H1A—C1—H1C	109.5	N3—C4—S1	129.07 (19)
H1B—C1—H1C	109.5		

### Hydrogen-bond geometry (Å, º)

**Table:**

*D*—H···*A*	*D*—H	H···*A*	*D*···*A*	*D*—H···*A*
N1—H1*D*···S1^i^	0.86	2.50	3.270 (2)	150
N3—H3*A*···N2^ii^	0.86	2.08	2.914 (3)	162

Symmetry codes: (i) *x*−1, *y*, *z*; (ii) *x*+1, *y*, *z*.
